# Impact of bowel resection margins in node negative colon cancer

**DOI:** 10.1186/s40064-016-3650-y

**Published:** 2016-11-11

**Authors:** Ricardo Rocha, Rui Marinho, David Aparício, Marta Fragoso, Marta Sousa, António Gomes, Carlos Leichsenring, Carla Carneiro, Vasco Geraldes, Vítor Nunes

**Affiliations:** B Surgery Department, Hospital Prof. Doutor Fernando Fonseca, Estrada IC-19, 2720-276 Amadora, Portugal

**Keywords:** Colon cancer, Colorectal surgery, Disease-free survival, Recurrence

## Abstract

**Purpose:**

Surgical intestinal resection margins in colon cancer are a longstanding debate in terms the optimal distance between the tumor and the colonic section line. The aim of this study is to define the oncological outcomes in relation to surgical margins, measured in terms or recurrence rate, time-to-recurrence, disease-free survival and overall survival in a population of node negative colon cancer patients.

**Methods:**

We conducted a retrospective observational longitudinal single institution study. All patients submitted to colon cancer surgery between January 2006 and December 2010 were analyzed. Only node negative patients were included in the study, with analysis of 215 patient charts, divided in two groups (Intestinal margin lower than 5 cm—group 1; and 5 cm or higher—group 2).

**Results:**

Mean age of patients was 70.4 years (±11.7), with a male predominance (57.7%). Group 2 more frequently corresponded to Stage II (83 vs 71%; p = 0.05). Global mean total lymph nodes harvested were 12, and were higher in group II than in group I (13.8 ± 8.2 vs 10.4 ± 5.7; p = 0.001). In terms of time-to-recurrence patients of group 2 had longer time than patients of group 1 (32.3 ± 12.1 vs 21.8 ± 13.8 months; p = 0.03), as well as a lower recurrence rate in group I (13.7 vs 17.2%), despite not statistically significant.

**Conclusions:**

This study has showed that patients with 5 cm or higher bowel resection margins had longer time-to-recurrence that was statistically significant. Recurrence rates were lower in the group of patients with longer surgical margins, however not statistically significant.

## Background

Historically it has been defined that intestinal resection margins in colon cancer should be 5 cm, on both sides of the tumor (Nelson et al. 2001). Evidence for this recommendation derive from clinical studies, in which surgical margins higher than 5 cm did not appear to decrease anastomotic recurrences (Devereux and Deckers 1985). However, anatomical studies have found that lymphatic tumor spread may be found at pericolic lymph nodes as far as 8–10 cm from the tumor, both sides (Morikawa et al. 1994; Toyota et al. 1995).

The recent description of complete mesocolic excision by Hohenberger et al., has allowed the standardization of colon cancer surgery as is done with Total Mesorectal Excision (Hohenberger et al. 2009). Central vascular tie and sharp separation of the visceral plane from the parietal plane have been defined as essential in order to achieve higher quality surgical specimens and better oncological outcomes (Hohenberger et al. 2009; West et al. 2014; Stelzner et al. 2016). Hohenberger et al. define surgical intestinal margins as 8 cm, in order to remove all pericolic lymph nodes involved in the tumor spread route (Hohenberger et al. 2009), however, there is lack of clear evidence to support this strategy.

It is unclear whether compromised surgical margins may lead merely to anastomotic recurrence or also to all kinds of recurrence. Theoretically, if we assume that tumor lymphatic drainage may involve lymph nodes in a length range of 8–10 cm, we should also assume that insufficient margins may lead to a subsequent insufficient lymphadenectomy that may lead to local, regional and even distant metastases.

On the other hand, node negative colon cancer patients still have recurrence rates that range from 5 to 25%, which has been related mainly to pathological tumor characteristics, but may also be avoidable with better quality surgery.

Our aim is to define the oncological outcomes in relation to surgical margins, measured in terms or recurrence rate, time-to-recurrence, disease-free survival and overall survival in a population of node negative colon cancer patients.

## Methods

### Study population

We retrospectively reviewed medical charts of 549 patients with primary adenocarcinoma of the colon submitted to surgery between January 2006 and December 2010 in our unit. The inclusion criterion was patients with curative intent resection with pathological node negative specimens. Exclusion criteria were as follows: metastatic disease at presentation; patients submitted to total colectomy and patients with known familial adenomatous polyposis. We recruited for the study population 215 patients.

### Data collected

Data on demographic parameters, histopathological examination, adjuvant treatment and recurrence were gathered for analysis in a standardized data collection form.

Demographic parameters included age, gender and co-morbidities.

Histopathological examination data included TNM classification, number of lymph nodes retrieved, lymphovascular invasion, perineural invasion, mucinous characteristics, histological differentiation, surgical intestinal longitudinal margins, perimeter and size of the tumor.

Patients were divided according to the intestinal longitudinal margins in two groups, lower than 5 cm and 5 cm or higher. The shorter longitudinal margin was the chosen one to register.

### Definitions

Locoregional recurrence comprised any disease reappearance at anastomotic site, retroperitoneal, mesenteric and peritoneal disease more than 6 months after surgery (reappearance of disease before this period was considered to be persistence and not considered for this study).

Distant metastases comprised any evidence of disease at distance, as lung, liver, bone, brain or distant lymph nodes according to the TNM classification REF.

Emergency presentation was defined as bowel obstruction or perforation leading to surgery within 24 h after admission.

Time-to-recurrence was defined as the length of time (in months) between surgery and any type of recurrence of the disease, meaning that the survival time of patients that did not recur are not considered.

Disease free-survival was defined as the length of time (in months) after surgery that the patient survives without any signs or symptoms of that cancer. This is a mean of the survival time without disease of all patients—for those with recurrence it is the time until recurrence and for those patients without recurrence is the total time of survival.

Overall survival was defined as the length of time between surgery and death of the patient, in months, regardless of the cause of death.

Surgical margins were measured after surgical specimen formalin fixation for pathological examination.

### Follow-up

Patients were followed up for at least 5 years postoperatively. Patients were scheduled for follow up every 3 months for the first year, every 6 months for the first 3 years and annually thereafter. Follow up evaluations included physical examination, CEA and CT scan. A colonoscopy was performed every 2 years. PET CT or liver MRI were performed according to clinician decision.

### Statistical analyses

Chi squared test and Fisher’s exact test were used to study categorical variables, ANOVA and logistic regression to study categorical and continuous variables and linear regression to study continuous variables. The Kaplan–Meier method was applied for survival analyses. To compare the survival distributions of two samples, the log-rank test was performed. For the analyses of disease-free survival, the first occurrence of locoregional recurrence, distant metastasis, or death due to a disease related cause was defined as an event. For the identification of observed survival, death due to any cause was defined as an event. A p value of less than 0.05 was appreciated to be significant. All analyses were performed using the IBM SPSS Statistics 23.0 software (SPSS Inc., Chicago, IL. USA).

## Results

### Patient characteristics

Regarding the population studied, the mean age was 70.4 years (standard deviation (SD) of 11.7), with a male predominance (57.7% male vs 42.3% female patients). Assessment by the type of surgery performed revealed that 36.3% of patients were submitted to right colectomy (78 patients), 1.9% to transverse colectomy (4 patients), 22.3% to left colectomy (48 patients) and 39.5% to high anterior resection for sigmoid cancer (85 patients).

Histopathologic examination showed that 22.3% of tumors had mucinous characteristics of some extent (48 cases); lymphovascular invasion was present in 14% (30 patients), well differentiated tumors represented 23.3% (50 cases), 61.4% were moderately differentiated (132 cases) and 14% were poorly differentiated (30 cases). The mean total lymph nodes harvested were 12 (SD 7). The mean tumor length was 4.6 cm (SD 2.2). The mean length of colon removed was 20.9 cm (SD 10.3 cm) and the mean length of small bowel resected was 10.3 cm (SD 5.6 cm) in right colectomies.

When looking at the shorter intestinal margin the mean length was 5.37 cm (SD 3.6 cm); with 7.5 cm (SD 3.95) for right colectomies, 2.85 cm (SD 1.18) for transverse colectomies; 4.9 cm (SD 3.13) for left colectomies and 3.8 cm (SD 2.52) for high anterior resections. This differences between the length of intestinal margins were statistically significant (p = 0.000).

Regarding AJCC tumor staging, 24.2% of patients (52 patients) corresponded to Stage I (T1 or T2) and 75.8% (163 patients) to Stage II (T3 or T4) disease.

Adjuvant chemotherapy was used in 16.7% of patients, corresponding mainly to stage II patients with features of high risk of recurrence (poorly differentiated, lymphovascular invasion, perineural invasion, presentation with perforation or bowel obstruction and less than 12 lymph nodes examined).

Respecting recurrences, we observed a global recurrence rate of 15.6% (34 patients), of which 6.5% (14 patients) had local recurrence, 7% (15 patients) had distant metastases and 2.3% (5 patients) had both.

We performed univariate analysis with all potential risk factors for recurrence (gender, age, type of surgery, elective vs emergent surgery, lymphovascular invasion, tumor differentiation, total lymph nodes harvested, colon length, bowel surgical margin and AJCC Stage) and the only that proved to be a risk factor for recurrence was lymphovascular invasion (12.3% of recurrences in patients without lymphovascular invasion vs 26.5% for those with lymphovascular invasion; p = 0.05).

Mean time-to-recurrence was 25.53 months (SD 13.9) and overall survival measured at 60 months was 53.7 months (SD 13.8).

**Fig. 1:**
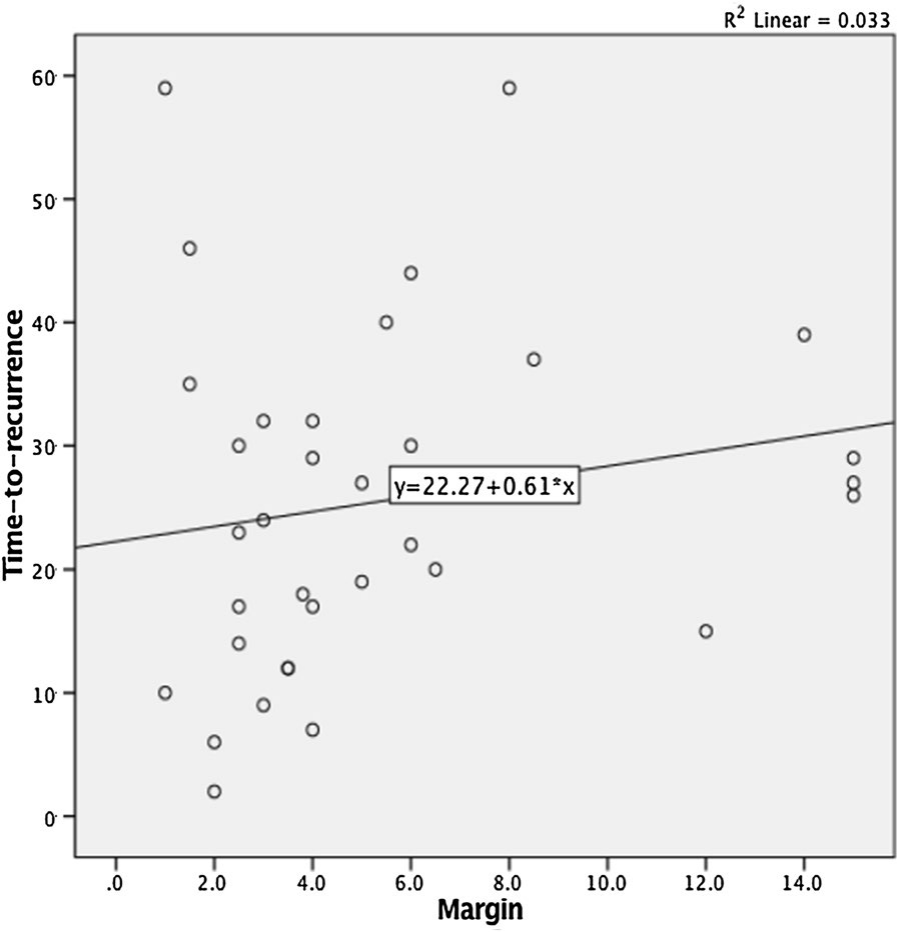
Linear relation between margin and time-to-recurrence

Bowel resection margins were linearly associated to time-to-recurrence (r^2^ 0.033; p = 0.1) (Fig. 1), as was the total number of lymph nodes harvested (r^2^ 0.102; p = 0.06). So, higher resection margins and higher lymph nodes yields were associated to larger time-to-recurrences.

On the other hand, longer resection margins were also linearly associated to a higher number of lymph nodes retrieved (r^2^ 0.102; p = 0.000), meaning that longer resection margins predisposed to higher number of lymph nodes in the specimen.

### Comparison between groups according to intestinal margin

The population was divided in two groups according to the shortest intestinal margin, group 1 being lower than 5 cm, and group 2 being 5 cm or higher (Tables 1, 2).

**Table 1:** Patient characteristics according to intestinal margins

	Lower than 5 cm	5 cm or higher	Odds ratio	P value
215 patients	128 (59.5%)	87 (40.5%)	–	–
Gender	Male—74 (58%)	Male—50 (57%)	1.014	1
	Fem.—54 (42%)	Fem.—37 (43%)		
Age	70.2 (SD ± 12.2)	70.8 (SD ± 10.9)	0.996	0.7
Type of surgery				0.000
Right colectomy	26 (20.3%)	52 (60%)	1.76	
Transverse colect.	4 (3%)	0 (0%)	1	
Left colectomy	29 (22.7%)	19 (21.8%)	1.048	
High AR	69 (53.9%)	16 (18.4%)	5.19	
Elective/emergent	112 (87.5%)/16 (12.5%)	68 (78%)/19 (21.8%)	0.511	0.09
Histopathologic features				
Lymphovascular invasion (Y)	19 (15%)	11 (13%)	1.21	0.69
Tumor differentiation (WD/MD/PD)	32 (25%)/79 (61.7%)/15 (11.7%)	18 (20.9%)/53 (61.6%)/15 (17.4%)	1.278 1.03 0.637	0.513
Total lymph nodes harvested	10.4 (SD ± 5.7)	13.8 (SD ± 8.2)	1.26	0.001
Colon length (in cm)	18.9 (SD ± 9.8)	23.7 (SD ± 10.3)	1.38	0.002
Intestinal margin (in cm)	3.1 (SD ± 1.2)	8.8 (SD ± 3.3)	*	0.000
Tumor length (in cm)	4.47 (SD ± 2.14)	4.69 (SD ± 2.3)	1.008	0.499
AJCC stage (I/II)	37 (29%)/91 (71%)	15(17%)/72(83%)	0.5	0.05

**Table 2:** Oncological outcomes according to intestinal margins

	Lower than 5 cm	5 cm or higher	Odds ratio	P value
215 patients	128 (59.5%)	87 (40.5%)		
Total recurrences	22 (17.2%)	12 (13.7%)	1.347	0.567
Locoregional	13 (10.1%)	6 (6.9%)	1.58	0.466
Distant metastases	12 (9.4%)	8 (9.2%)	1.07	1
Time-to-recurrence (in months)	21.8 (SD ± 13.8)	32.3 (SD ± 12.1)	0.9	0.03
Overall survival (in months)	52.9 (SD ± 14.7)	54.9 (SD ± 12.3)	0.98	0.314

The two groups were statistically similar in terms of gender (male gender in group 1 58 vs 57% in group 2; p = 1), age (mean 70.2 years vs 70.8; p = 0.7), emergent surgery (12.5 vs 21.8%; p = 0.09), lymphovascular invasion (15 vs 13%; p = 0.69), tumor differentiation and tumor length (4.47 vs 4.69 cm; p = 0.499) (Table 1).

The two groups were significantly different in what concerned the type of surgery (Table 1), being the right colectomies more frequently associated with higher intestinal margins and the sigmoid cancer surgery more frequently associated with shorter margins. Higher surgical margins were also associated with higher lymph nodes retrieval as well as to longer length of colon specimen resected (Fig. 1).

Patients with higher surgical margins were those with the higher AJCC stage, with a stronger predominance of T3 tumors (66.7%).

Regarding the oncological outcomes, although the group of patients with bowel margins higher than 5 cm had a tendency to lower global recurrence (13.7 vs 17.2%), lower locoregional recurrences (6.9 vs 10.1%) and lower distant metastases rates (9.2 vs 9.4%), this wasn´t statistically significant (Table 2).

**Fig. 2:**
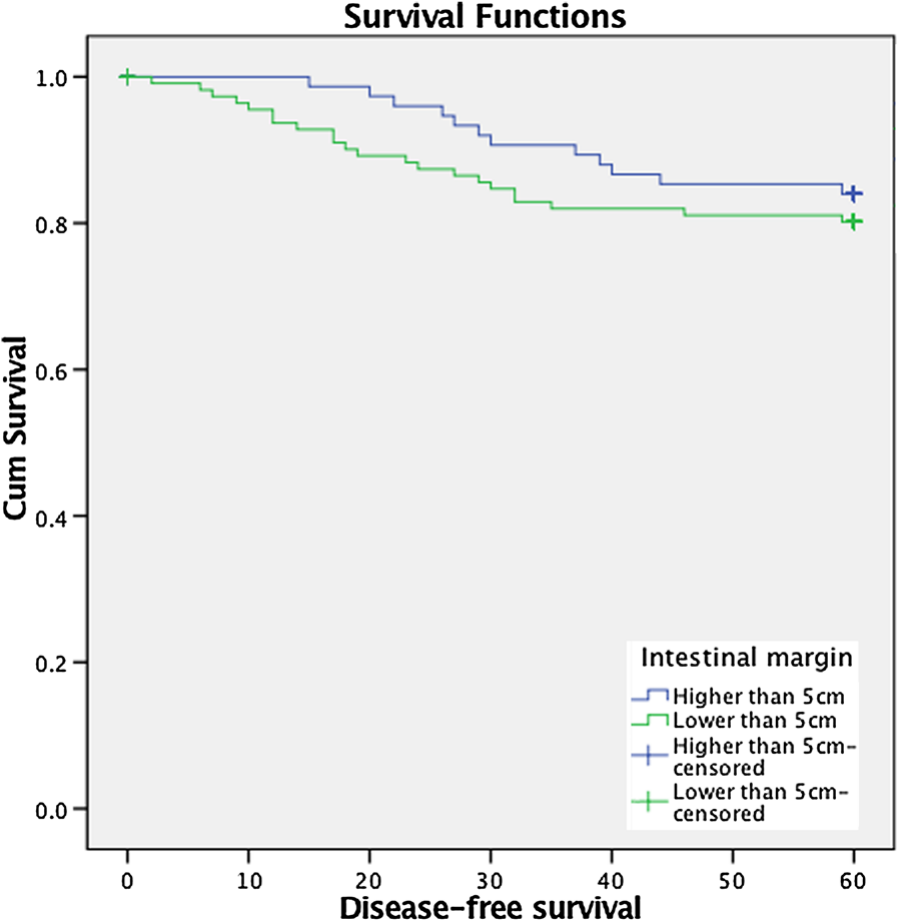
Disease-free survival according to intestinal margin

In terms of time-to-recurrence, patients of group 2 (higher than 5 cm) had longer time to recurrence than patients of group 1 (32.3 vs 21.8 months; p = 0.03) and had longer disease-free survival (log-rank test 0.6; p > 0.05) (Fig. 2) though not statistically significant. Therefore, patients with shorter margins had earlier recurrences than the group of patients with larger margins.

## Discussion

The optimal bowel proximal and distal surgical margins are an interesting and longstanding debate. Historically, 5 cm macroscopic margin has been considered, measured intra-operatively by the surgeon, as the optimal margin, based on anatomical and clinical studies (Devereux and Deckers 1985; Morikawa et al. 1994; Toyota et al. 1995).

However, recent studies have demonstrated that pericolic lymph nodes as far as 8–10 cm of the tumor may harbor tumor metastases, questioning traditional colonic resection margins in colon cancer surgery (Stelzner et al. 2016). Complete mesocolic excision description has standardized colon cancer surgery focusing on the concepts of sharp dissection, surgical mesocolic plane, mesocolic integrity, high vascular tie, distance between the tumor and the vascular ligation and length of colon resected (West et al. 2010).

In this retrospective study comparing oncological outcomes according to surgical intestinal resection margins we found that the two groups (<5 and ≥5 cm) weren´t different in terms of gender, age, setting in which surgery was performed (elective vs emergent), lymphovascular invasion, tumor differentiation and tumor length (Table 1).

The two groups were statistically different regarding the type of surgery performed, total lymph nodes harvested and AJCC stage (Table 1).

The group of patients with intestinal margins 5 cm or higher was more likely to have T3/T4 tumors (AJCC Stage II). Though this locally advanced tumors might have a higher probability of recurrence, this wasn´t the case in our study population, in which they had a tendency to lower global recurrence, however without statistical significance. This might translate a surgeon´s tendency to do a wider resection in more locally advanced tumors and that this wider surgery with more lymph nodes retrieved might counterbalance the worst inherent prognosis of this more advanced disease.

We observed an overall 5-year recurrence rate of 15.6%, which is comparable to other studies of node negative colon cancer that may be up to 25%. The overall survival at 5 years was 72.5%, also comparable to the literature, in which overall survival ranges between 80 and 95% in stage I and 65–75% in stage II (Weitz et al. 2005). Intestinal resection margins may be one of the factors responsible for this variability.

Despite no statistically significant difference between recurrence rates, we found in our study that there was a significant difference in time-to-recurrence between the groups. Patients with intestinal margins shorter than 5 cm had more early recurrences than patients with intestinal margins 5 cm or higher (21.8 vs 32.3 months).

Margin measurements were performed after formalin fixation, which means that actual bowel resection margins were superior to those stated in this paper. In fact, bowel margins measurement has always raised important issues. Studies often fail to specify when measurements were made, intra-operatively or after fixation, which may lead to different conclusions (Weese et al. 1986). Indeed, it has been proven that surgical margins vary considerably according to this criterion. Experimental animal studies have shown that immediately after excision the bowel length suffers a mean shrinkage of 28.3%, and an additional 26.3% after 24 h of formalin fixation (Clarke et al. 2014). Even before formalin fixation, immediately after the resection there is a loss of colon length and contractility that interfere with margin measurements, responsible for a 15.6% variation (Wang et al. 2004).

Intestinal margins probably also affect lymph node retrieval, which may have staging repercussion and, more importantly, oncological consequences. Recent papers have stressed that increasing lymph node retrieval in node negative cancers has a positive impact in survival, albeit without improvements in tumor staging by current standard histological analysis (Weitz et al. 2005; Gleisner et al. 2013; Budde et al. 2014). It has been stated that higher lymph node retrieval in node negative patients, up to 25 lymph nodes, decreases the risk of death (Weitz et al. 2005).

We have shown that increasing intestinal margins linearly increases the total number of harvested lymph nodes and that intestinal margins as well as lymph node harvested have a linear correlation with time-to-recurrence, meaning that larger margins and higher number of lymph nodes retrieved prolong the time-to-recurrence in patients that will eventually recur.

In our study the overall 5 years survival was slightly higher in patients with margins superior to 5 cm, however this wasn´t statistically significant. On the other hand, Kaplan–Meyer survival curves demonstrated the benefit in terms of disease-free survival and time-to-recurrence in patients that have longer surgical intestinal margins, despite not being statistically significant.

Regarding the type of surgery performed we realized that high anterior resections for sigmoid colon cancer were more likely to have shorter margins than right and left colectomies, with an OR of 5.19 (p = 0.000), this might pose the question of the importance of real high ligation of the IMA as described by Hohenberger (Hohenberger et al. 2009).

This study had some limitations. It was a single institution retrospective study, surgeries were performed by several surgeons and the margins were measured after formalin fixation, which may introduce variability in margin status.

We have shown that surgical resection margins in colon cancer surgery has an impact on oncological results namely time to recurrence. This study is one more contribute to further clear the question of correct surgical margins in the path of colon surgery standardization introduced by complete mesocolic excision.
